# Induction of apoptosis in HeLa cells by chloroform fraction of seed extracts of *Nigella sativa*

**DOI:** 10.1186/1475-2867-9-29

**Published:** 2009-11-27

**Authors:** Gowhar Shafi, Anjana Munshi, Tarique N Hasan, Ali A Alshatwi, A Jyothy, David KY Lei

**Affiliations:** 1 Molecular Cancer Biology Lab, Department of Food Science and Nutrition, King Saud University, Riyadh, Saudi Arabia; 2 Department of Molecular Biology, Institute of Genetics and Hospital for Genetic Diseases, Hyderabad, India; 3 Department of Nutrition and Food Science, University of Maryland, College Park, Maryland, USA

## Abstract

**Background:**

Cancer remains one of the most dreaded diseases causing an astonishingly high death rate, second only to cardiac arrest. The fact that conventional and newly emerging treatment procedures like chemotherapy, catalytic therapy, photodynamic therapy and radiotherapy have not succeeded in reverting the outcome of the disease to any drastic extent, has made researchers investigate alternative treatment options. The extensive repertoire of traditional medicinal knowledge systems from various parts of the world are being re-investigated for their healing properties. This study progresses in the direction of identifying component(s) from *Nigella sativa *with anti cancer acitivity. In the present study we investigated the efficacy of Organic extracts of *Nigella sativa *seed powder for its clonogenic inhibition and induction of apoptosis in HeLa cancer cell.

**Results:**

Methanolic, n-Hexane and chloroform extracts of *Nigella sativa *seedz effectively killed HeLa cells. The IC_50 _values of methanolic, n-hexane, and chloroform extracts of *Nigella sativa *were 2.28 μg/ml, 2.20 μg/ml and 0.41 ng/ml, respectively. All three extracts induced apoptosis in HeLa cells. Apoptosis was confirmed by DNA fragmentation, western blot and terminal transferase-mediated dUTP-digoxigenin-end labeling (TUNEL) assay.

**Conclusion:**

Western Blot and TUNEL results suggested that *Nigella sativa *seed extracts regulated the expression of pro- and anti- apoptotic genes, indicating its possible development as a potential therapeutic agent for cervical cancer upon further investigation.

## Background

Around one quarter of deaths in the US are accounted for by Cancer. According to the American Cancer Society, on an average, 559,312 people die of the disease each year [1] despite tremendous efforts to find methods of control and cure. Thus, not surprisingly, every fourth citizen of a developed country will be stricken sometime during his/her life and approximately 400 new incidents emerge per 100,000 people annually [2]. The statistics released by WHO in 2008 and GLOBOCAN indicate that there is a high likelihood of developing countries approaching the same incident rates of cancer as developed ones, because of life style changes, average age of the population, tobacco usage, etc [3,4]. In a scenario where conventional medicine has failed to develop techniques that could reduce the incidence of death due to cancer, complementary and alternative medicine (CAM) is slowly emerging as an option. A variety of ingredients of traditional medicines and herbs are being widely investigated in several parts of the world to analyze their potential as therapeutic agents [5-7]. According to the National Institute of Health's National Centre for Alternative and Complementary medicine, around 36% of people in the US use alternative medicine in some form or the other. Several studies indicate that a majority of cancer patients use CAM extensively as a mode of treatment or as a means to reduce the side effects of conventional treatment methods [8-10].

There are a number of alternative medicine systems based on traditional theories and philosophy that have originated in specific geographical areas and evolved over the years. The most widely practiced of these include the *Unani, Ayurveda & Siddha *and the *Chinese *system of medicine that have originated from the Arab, India and China respectively. These medicinal systems have identified more than 700 individual herbal extracts as well as several drug preparations which claim to treat and/or prevent several diseases including cancer [11].

Apoptosis an active physiological process resulting in cellular self-destruction of unwanted cells is absent in cancer cells. Apoptosis is characterised by distinct morphologic changes, including cell shrinkage, membrane blebbing, chromatin condensation, DNA fragmentation, and the formation of apoptotic bodies [12]. Bcl-2 family proteins determine whether a cell lives or dies, by controlling the release of mitochondrial apoptogenic factors, which are associated with death proteases, called caspases [13]. Caspases, a class of cysteine proteases, are central players in the apoptotic process that trigger a cascade of proteolytic cleavage events [14]. The activation of caspase-3 is an important downstream event in apoptosis [15]. p53, a tumor suppressor gene, helps regulate the cell cycle. It plays a key role in ensuring that damaged cells are destroyed by apoptosis (programmed cell death). p53 is the most commonly mutated gene associated with cancer. The regulation of apoptosis in normal and malignant cells has become an area of intensive study in cancer research [16]. The therapeutic application of apoptosis is currently being considered as a model for the development of anti-tumour drugs [17]. It is therefore essential to identify novel apoptosis-inducing compounds that are candidate anti-tumour agents.

In present study we chose to investigate the extracts of *Nigella sativa *seed (NS) for testing its in vitro apoptotic activity. It is an annual herb belonging to the family Ranunculaceae and is commonly known as black cumin and is a natural food additive in India and many parts of Asia. The seeds of NS are the source of active ingredient of this plant. In the *Unani *system, it is regarded as one of the greatest forms of healing medicine available.

The bioactivity of the NS seeds has been investigated both in vitro as well as in vivo for a number of therapeutic properties like antioxidant, antihistaminic, anti inflammatory, immune-modulatory, anti viral, anti helimenthic, anti bacterial [18] and its clononogenic effect on PC-3 cells [19]. However, its anti tumor properties have to be researched further.

Cervical cancer forms an important part of cancer sub-types among women and is more common in Hispanic and African-American women than in Caucasians. It is the fifth most common type of cancer among women with 471,000 new cases each year with 11,070 cases in US in 2008 and 3,870 deaths according to the data released by the National Cancer Institute. Considering the significance of this cancer sub-type accounting to a death every 2 minutes, we used HeLa cell lines to analyze the anticancer activity of the NS seed extract and sought to investigate the ability of NS to induce apoptosis and to determine the biochemical mechanisms underlying apoptosis in HeLa cells. To determine the species' antiproliferative activity, we examined the effects of various solvent fractions on cell viability by clonogenic inhibition assay. Induction of apoptosis was assessed by DNA fragmentation, TUNEL assay and western blot for the expression of the anti-apoptotic protein Bcl-2 & bcl-X_L _and of the pro-apoptotic proteins caspase-9, caspase-8 and caspase-3 and p53. The results led us to hypothesize that certain bioactive compounds in NS seeds might be capable of effectively causing death of cancer cells by up-regulating the expression of certain pro-apoptotic gene and simultaneously down-regulating the expression of anti-apoptotic genes.

## Results and Discussion

### NS extract-induced clonogenic inhibition of HeLa cells

**Figure 1:**
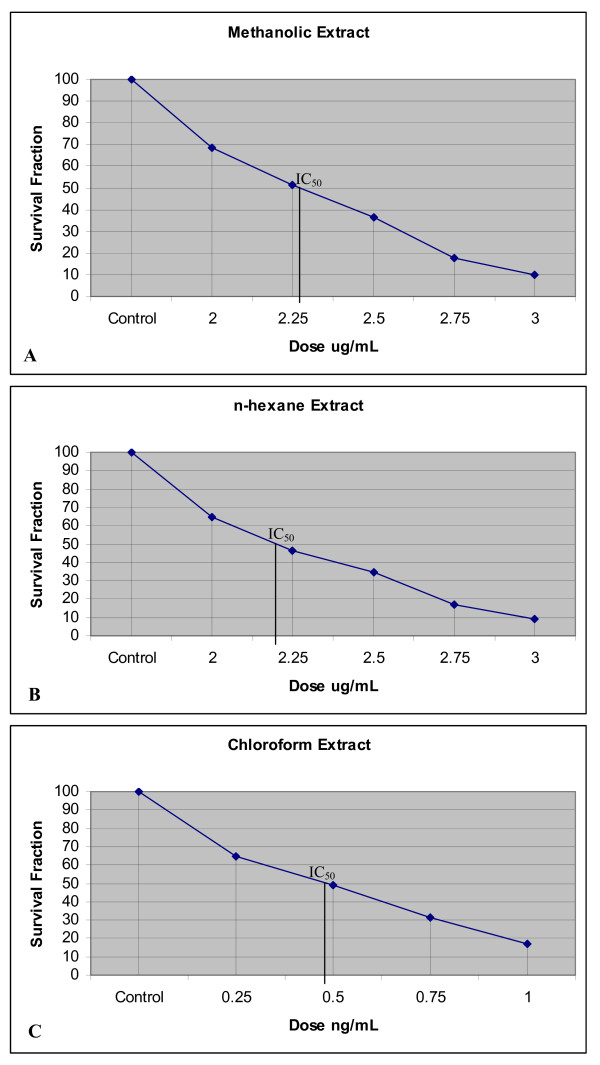
**A NS-Methanolic Extracts induced Clonogenic inhibition in HeLa cells**. **1B**. NS n-haxane Extracts induced Clonogenic inhibition in HeLa cells. **1C**: NS-Chloroform Extract induced Clonogenic inhibition in HeLa cells

The methanolic extract (IC_50 _2.28 μg/mL) (Fig 1A), n-hexane extract (IC_50 _2.20 μg/mL), (Fig 1B) and chloroform extract (IC_50 _0.41 ng/mL) (Fig 1C) significantly inhibited the growth of HeLa cells. Further, all three extracts (n-hexane, methanolic and chloroform) produced significant clonogenic inhibition of HeLa cells. The IC_50 _values clearly indicated that the chloroform extract had a much more potent effect on HeLa cells than the n-hexane and methanolic extracts.

### Quantification of Apoptosis by DNA fragmentation and TUNEL assay

**Figure 2:**
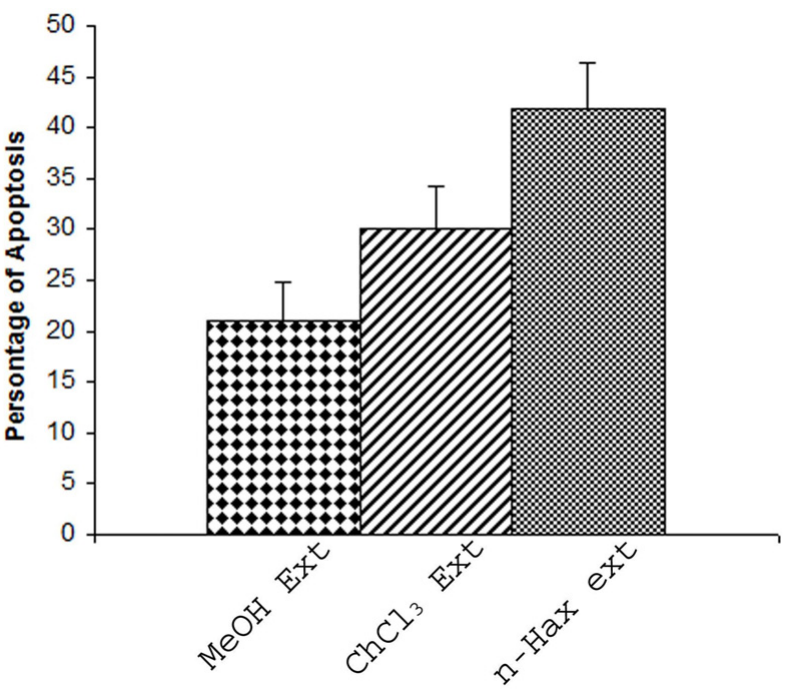
**NS extracts induced apoptosis in HeLa cells**. The bar graphs show the percentage of TUNEL-positive cells. Apoptosis in the treated cells was normalized to the baseline apoptosis in the untreated cells. The data shown are the means from two independent experiments.

**Figure 3:**
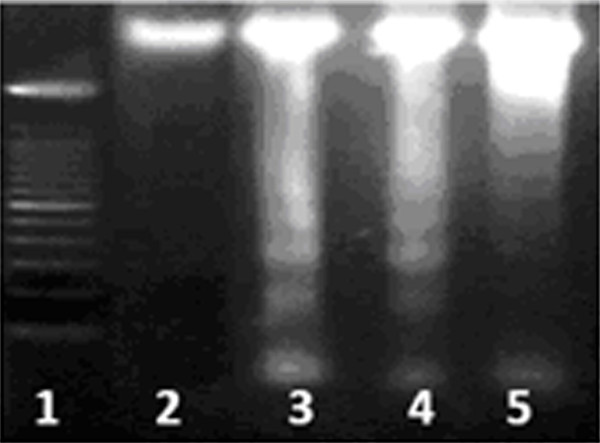
**DNA laddering**: DNA fragmentation on 1% Agarose Gel Electrophoresis shows; Lane 1 100 bp marker, Lane 2 untreated cells, Lane 3 Chloroform extract treated cells, Lane 4 n-hexane extract treated cells and Lane 5 methanolic extract treated cells.

Apoptosis is a form of programmed cell death characterized by cytoplasmic condensation, plasma membrane blebbing and nuclear pycnosis, leading to nuclear DNA breakdown into multiples of ~200 bp oligonucleosomal size fragments. The detection of apoptosis in cultured cells relies heavily on techniques involving the extraction of nuclear DNA and characterization of such oligonucleosomal ladders by gel electrophoresis and detection of DNA damage by end labeling using terminal transferase-mediated dUTP-digoxigenin-end labeling (TUNEL). HeLa cells were treated with respective IC_50 _concentration of NS seed extracts as follows: chloroform extract at 0.41 ng/ml, the n-hexane extract at 2.20 μg/ml and the methanolic extract at 2.28 μg/ml to determine whether the bioactive compounds of NS seeds induced HeLa cell death via apoptosis or by necrosis. HeLa cells treated with these extracts showed active apoptosis after 24 hours of treatment; the increase in apoptosis over the untreated population was 21.1% in methanolic extract treated cells, 30% n-hexane extract treated cells and 42% chloroform extract treated cells (Fig 2). Apoptosis was determined by microscopic based TUNEL examination and DNA fragmentation (Fig 3).

### Altered Apoptotic Gene Activity and Caspase activation by NS Seed extract treatment in HeLa Cells

**Figure 4:**
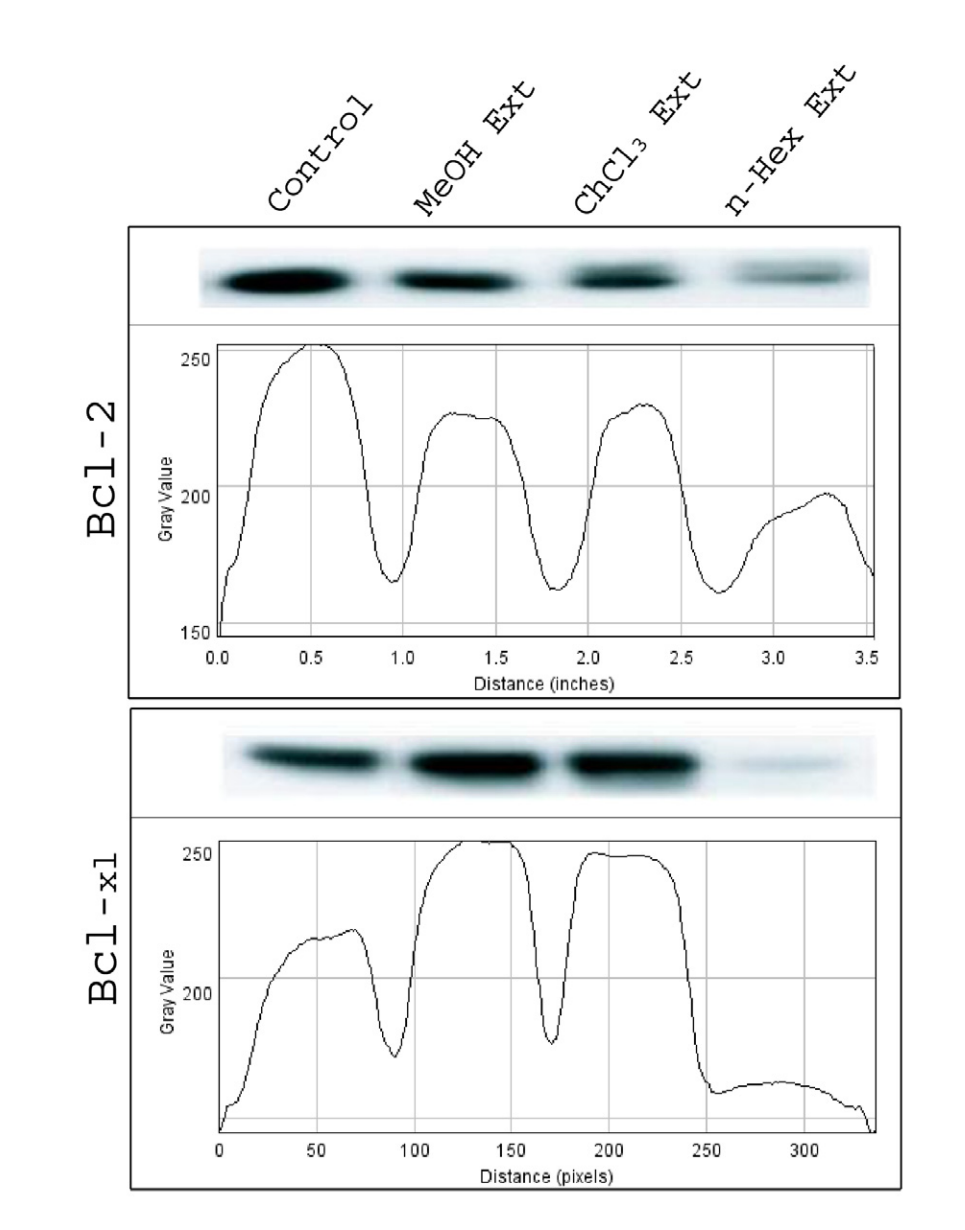
**Expression of anti-apoptotic genes; bcl-2 and bcl-X_L _in HeLa cells treated with methanolic, choloroform and n-haxane extract**. The graph shows the intensity of bands (ImageJ sofware).

**Figure 5:**
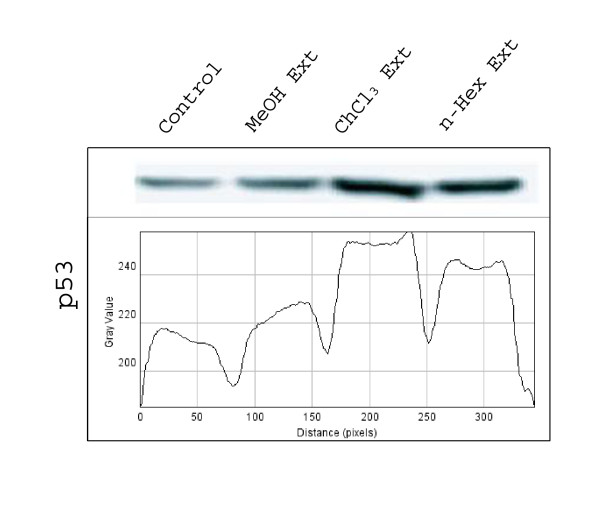
**Expression of caspase -3, -8 and -9 in HeLa cells treated with methanolic, choloroform**. and n-haxane extract. The graph shows the intensity of bands (ImageJ sofware).

**Figure 6:**
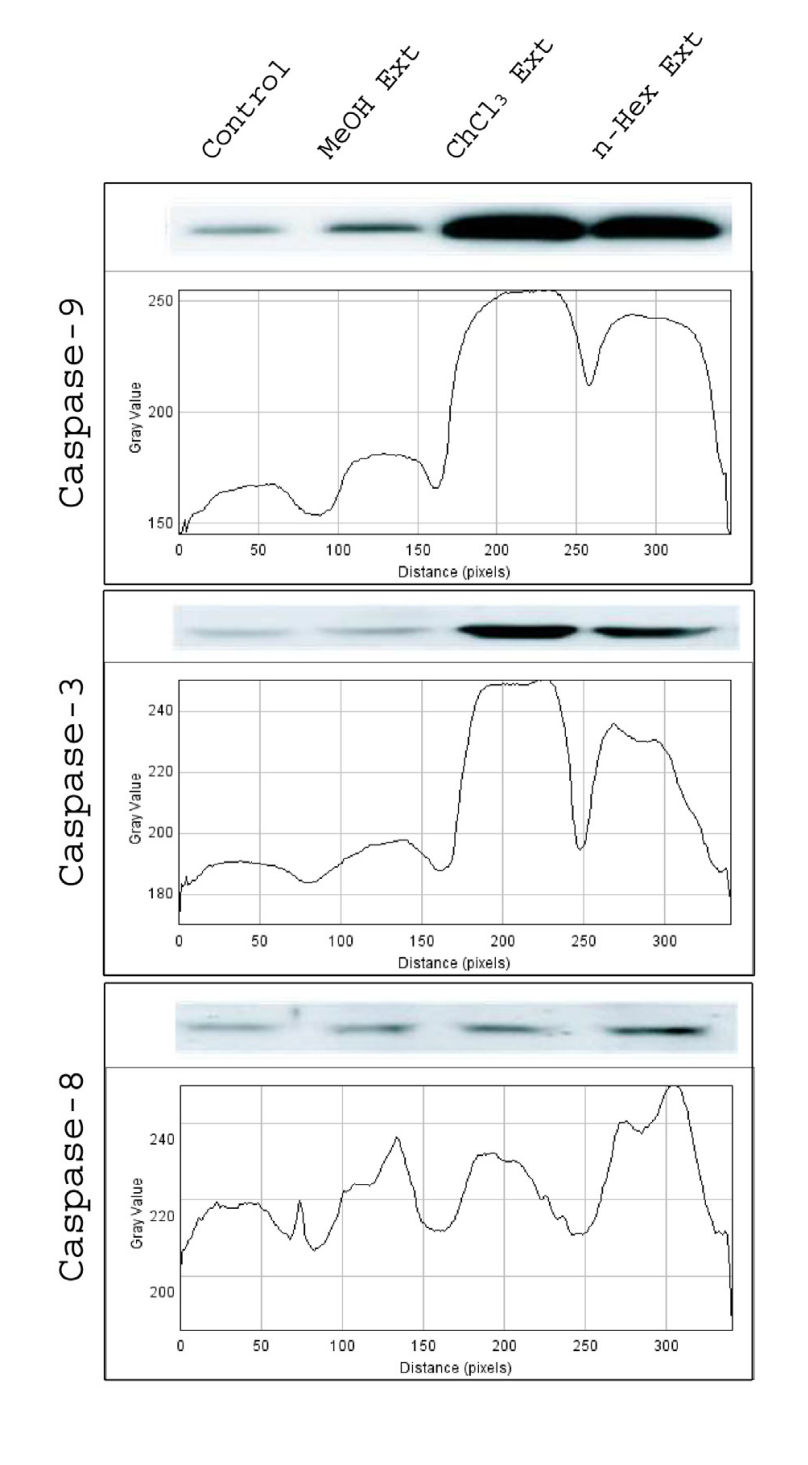
**Expression of pro-apoptotic gene, p53 in HeLa cells treated with methanolic, choloroform**. and n-haxane extract. The graph shows the intensity of bands (ImageJ sofware).

To determine the mechanism of NS-induced apoptosis, the expression of anti- and pro-apoptotic proteins following NS treatment was examined by western blotting. It was clear from western blot results that the expression of *p53*, *caspase-3*, *-8 *and *-9 *was higher in chloroform extract treated cells, followed by n-hexane and methanolic extracts treated cells compared to respective controls. However *bcl-2 *and *bcl-X*_*L *_were down regulated in n-hexane extract treated cells compared to chloroform and methanolic extract treated and untreated cells (Fig 4). This suggested that n-hexane contains some compounds which down regulates the expression of *bcl-2 *and *bcl-X*_*L*_. Similarly, chloroform, n-hexane and methanolic extracts also contained certain compounds which up regulate the expression of *p53 *(Fig 5), *caspase-3*,-*8 *and -*9 *(Fig 6).

## Conclusion

The drug discovery process is becoming more and more complex and capital-intensive, and it calls for systematic and critical review of methods and approach towards the entire process, a need to rediscover the discovery process afresh.

The Indian Systems of Medicine are in the list approved by the National Council for Alternative Medicine (NCAM), USA, although most of these drugs that claim to cure various ailments are yet to be validated scientifically. According to Parasnis [20], the full potential of Ayurveda can be realized only by subjecting the ayurvedic drugs to modern investigation techniques. Further acceptance of any clinical trial depends on whether it satisfies modern pharmacological and statistical standards. Unfortunately, most people practicing Ayurveda decline to adopt modern research techniques for evaluation of the formulations used in the practice. This holds good for other traditional medical practitioners like those of Siddha and Unani medicinal systems too. Scientific validation will not only popularize these medicines in India but also render them acceptable, in some form, to people in other parts of the world. Considering the fact that several diseases do not have an ultimate answer in the conventional system whether in native regions or throughout the world, an effort to recognize the potential of alternative and combinational treatment systems validated through universally acceptable methods could prove to be very beneficial for the human community at large.

Screening of medicinal plants for potential anticancer properties has increased greatly over the past few decades. For instance, the US National Cancer Institute has implemented a large-scale project of acquisition and screening of compounds isolated from medicinal plants originating in various parts of the world. These medicinal plants are identified based on ethnopharmacological, chemosystemic and ecological information.

Therefore, there is a need for more effective anticancer agents since the most common tumors in adults are resistant to almost all presently available anticancer drugs and the majority of the available drugs have limited anti-solid tumor activity [21].

Apoptosis is an active physiological process resulting in cellular self-destruction that involves specific morphological and biochemical changes in the nucleus and cytoplasm [22]. Agents that suppress the proliferation of malignant cells by inducing apoptosis may represent a useful mechanistic approach to both cancer chemoprevention and chemotherapy. While many anticancer agents have been developed, unfavourable side effects and resistance are serious problems [23]. Thus, there is growing interest in the use of plant materials for the treatment of various cancers and the development of safer and more effective therapeutic agents [24]. NS seeds have been used as a folk remedy for several diseases including cancer without any understanding of the underlying mechanisms. In this paper, we investigated the influence of NS seed on cellular proliferation and apoptosis using various extract fractions. The NS induced apoptosis in HeLa cells, indicating that it may be used as a therapeutic agent for human cervical cancer. In general, apoptosis is controlled by the complex interplay between regulatory proteins from the Bcl-2 family [25]. These pro- and anti-apoptotic proteins are key regulators of the intrinsic pathway of apoptosis, controlling the point of no return and setting the threshold for engagement of the death machinery [26]. Previous reports have shown that the ratio of Bax to Bcl-2 determines, in part, the susceptibility of cells to death signals [27]. Therefore, Bcl-2 proteins have emerged as an attractive target for the development of novel anticancer drugs [28]. Changes in the Bcl-2/Bax ratio have been reported to be caused by downregulation of Bcl-2 and slight downregulation of Bax [29], downregulation of Bcl-2 and upregulation of Bax [30,31], and downregulation of Bcl-2 with no change in the level of Bax [32]. In this study, we demonstrated that Bcl-2 and bcl_XL _expression was significantly inhibited while p53 and caspase -3,-8 &-9 expression was markedly increased in a concentration-dependent manner. Our results suggest that the NS induced apoptosis by regulating apoptotic genes.

The mechanism of action of many anticancer drugs is based on their ability to induce apoptosis [33,34]. Hence we were interested to identify if cancer cells treated with various organic extracts of *N. sativa *used apoptosis as their mode of cell death. This was investigated by studying distinct morphological features (nuclear chromatin condensation, fragmentation of nuclear material) and molecular features (expression of certain crucial genes).

The NS seed powder was subjected to three different organic extractions with increasing polarity *viz*. methanol, chloroform and n-hexane. This was to reduce the concentration of the NS seed components in each extract, so that the potent compound(s) can be identified. These extracts regulated the expression of specific target genes. It was found that the chloroform extract led to over expression of pro-apoptotic genes while n-hexane and methanolic extracts led to down regulation of anti-apoptotic genes when compared to respective controls. After sufficient analysis, we could conclude that the NS extracts contain potent component(s) which have the ability to induce apoptosis through caspase and *p53 *pathway in HeLa cells. However, further studies are required to find the exact compound(s) and their structure(s) as well as to find whether the same extract/compound(s) are also capable of regulating the expression of the same genes in other cancer cell types. In addition, it was observed that the n-haxane extract down- regulated the expression of bcl-2 and bcl-X_L _and the methanolic and chloroform extract inhibited the expression of bcl-2, though to a smaller extent.

Efforts towards finding a practical solution in combating this dreadful disease could prove to be of paramount importance. There is no paucity on the availability of umpteen numbers of medicinal plants known to regional communities practicing indigenous healing techniques, which can on scientific evaluation provide an alternative to the presently available options that do not go without severe and painful side-effects. Many herbs have been evaluated in clinical studies and are currently being investigated phytochemically to understand their tumouricidal action against the insidious nature cancer.

Ayurvedic therapy has been found to cure these chronic diseases like caner better, which are previously not amenable to treatment by western medical practices [35]. This traditional Indian medicine with its evolution through centuries has always fascinated practitioners and researchers for its applications in cancer treatment on a scientifically proven research background.

Herbal decoctions consisting of multiple herbs each possessing tremendous potential for a cancer cure are commonly used in Ayurveda. These formulations are reported to work on multiple biochemical pathways and are capable of influencing several organ systems simultaneously. The benefit of a herbal decoction is that it can nourish the body as a whole by supporting various organ systems. More importantly as it has no synthetic part to it, there is very less likelihood of an unexpected adverse effect. Many of the herbs have been scientifically-proven for anti-cancerous properties and are used for the treatment of various cancers [36,37]. They include the Vinca alkaloids (from the *Madagascar periwinkle*, *Vinca rosea*), irinotecan (from *Camptotheca accuminata*) and paclitaxol (from the Pacific yew tree *Taxus brevifolia*). Future research in this area would help in the identification of safe and effective anticancer drugs.

However, the potent extracts should be tested on several other cancer cell lines, biochemical and molecular studies carried out using the extracts in animal models to establish their therapeutic efficacy as well as toxicity, and the most potent extracts subjected to HPLC and GC&MS analyses to identify and characterize the efficacious phytotherapeutic and/or bioactive compound(s) in NS.

## Materials and methods

### Preparation of organic extract

**Figure 7:**
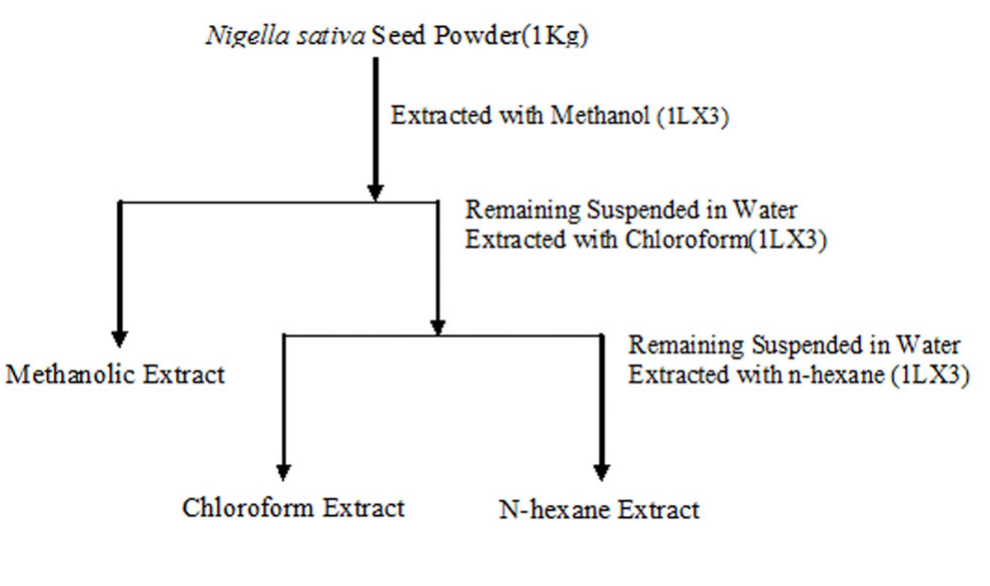
**Extraction and partitioning of bioactive compounds of *N. sativa***.

Air dried NS seeds were pulverized using a milling machine and extracted with Methanol, *n*-hexane, Chloroform, using the Soxhlet apparatus, as represented in the flow chart (Fig 7). Each organic phase was later evaporated under reduced pressure to obtain the residue, using a rotary evaporator to dryness in order to obtain the respective lyophilized powder/paste, weight and required quantity was dissolved in Dimethyl Sulfoxide (DMSO).

### Maintenance of HeLa cell line

HeLa cells were procured from the National Centre of Cell Sciences (NCCS), Pune, India. Cell lines were maintained and propagated in 90% Dulbecco's Modified Eagle's Medium (DMEM) containing 10% fetal bovine serum (FBS) and 1% penicillin/streptomycin. Cells were cultured as adherent monolayer and maintained at 37°C in a humidified atmosphere of 5% CO_2_. Cells were harvested after subjecting them to brief trypsinization. All Chemicals were research grade.

### Cell Viability Assay

Cell viability was assayed where and as required by trypan blue exclusion test [38] with slight modifications. The viability of cells was found to fall between 90-95%.

### Colonogenic Inhibition Assay

The Colonogenic inhibition assay was performed as described previously by us [19]. Briefly, two different cell concentrations in quadruplet sets were used for one dose point. After incubation for 13 days, each flask was stained with crystal violet and the colonies containing more than 50 cells were counted. The surviving fraction was calculated by the following equation, and then normalized by cell multiplicity:

(Where PE is the plating efficiency).

#### Determination of IC_50 _Concentration

The IC_50 _value (the concentration of the drug which is capable of bringing about 50% inhibition of colony formation) of the drug used for treatment was determined by plotting a graph with Survival fraction (Y-axis) against the concentrations of the extracts (X-axis) [Fig 1A, B &1C].

#### Analysis of DNA Fragmentation

HeLa cells were treated with respective concentration (IC_50_) of each extract for 24 hours and then harvested. Genomic DNA was extracted by standard salting out method with slight modification [21] and was electrophoresed on 1.8% agarose for DNA fragmentation which allows to determine the amount of DNA that is degraded upon treatment of cells with certain agents.

#### TUNEL Assay

To quantify apoptosis, the ApopTag™ in situ apoptosis detection kit (Oncor, Gaithersburg, MD) was used as per the instructions of the manufacturer. The ApopTag kit detects DNA strand breaks in single cells by terminal transferase-mediated dUTP-digoxigenin-end labeling (TUNEL). Briefly, cells were seeded in chamber slides and the next day they were exposed to each organic extracts. After 24 hrs the DNA was tailed with digozigenin-dUTP and cojugated with an anti-digoxigenin fluorescein. The cells were then counterstained with propidium iodide and antifade. The stained cells were observed using Carl-Zeiss epifluorescence microscope using triple band-pass filter. To determine the percentage of cells indicating apoptosis, a total of 1000 cells were counted in each experiment.

#### Western Blot Analysis

After treatment, the cells were collected and washed twice with cold PBS. The cells were then lysed in lysis buffer (50 mM Tris-HCl, pH 7.5, 150 mM NaCl, 1% Nonidet P-40, 2 mM EDTA, 1 mM EGTA, 1 mM NaVO3, 10 mM NaF, 1 mM DTT, 1 mM PMSF, 25 lg/ml aprotinin, and 25 lg/ml leupeptin) and kept on ice for 30 min. The lysates were then centrifuged at 12,000 g at 4°C for 20 min; the supernatants were stored at -70°C until use. The protein concentration was determined by the Bradford method. Aliquots of the lysates (30 μg of protein) were separated by 12% SDS-PAGE and transferred onto a nitrocellulose membrane using transfer buffer (192 mM glycine, 25 mM Tris-HCl, pH 8.8, and 20% methanol [v/v]). After blocking with 5% non-fat dried milk, the membrane was incubated for 2 h with primary antibodies, followed by 30 min with secondary antibodies in milk containing Tris-buffered saline (TBS) and 0.5% Tween. Anti-human p53, caspase-3, caspase-8, -caspase-9, -Bcl-2, and bcl-X_L _antibodies were used at a 1:1000 dilution as the primary antibodies, while horseradish peroxidase-conjugated horse anti-rabbit IgG (Sigma Chemicals, USA) was used at a 1:5000 dilution as the secondary antibody. The membrane was then exposed and protein bands were detected using Enhanced Chemiluminescence.

### Statistical analysis

The results of each series of experiments (performed in triplicates) are expressed as the mean values ± standard deviation of the mean (SD).

## Abbreviations

TUNEL: terminal transferase-mediated; dUTP: digoxigenin-end labeling; CAM: Complementary and alternative medicine; NS: *Nigella sativa*; NCCS: National Centre for Cell Science; DMSO: Dimethyl Sulfoxide; NCAM: National Council for Alternative Medicine; FBS: Fetal Bovine Serum; DMEM: Dulbecco's Modified Eagle's Medium;

## Competing interests

The authors declare that they have no competing interests.

## Authors' contributions

GS is a researcher working in cancer biology and carried the overall study. GS and TNH undertook the Statistical analysis. AM along with JA and DKYL designed the work and interpreted the results. AM, GS and AAA contributed to the writing of the manuscript, which was edited, revised critically and co-ordinated by DKYL. All the authors read and approved the final manuscript.
